# Utilisation of nursing home care before and after the 2015 Dutch national reform: an interrupted time series analysis

**DOI:** 10.1093/ageing/afaf018

**Published:** 2025-02-04

**Authors:** Janet L MacNeil Vroomen, Joost D Wammes, Bram Wouterse, Martin Smalbrugge, Terrence E Murphy

**Affiliations:** Internal Medicine, Section Geriatric Medicine, Amsterdam UMC Locatie Academic Medical Center, Amsterdam, Netherlands; Internal Medicine, Section Geriatric Medicine, Amsterdam UMC Locatie Academic Medical Center, Amsterdam, Netherlands; Ageing and Later Life, Amsterdam Public Health Research Institute, Amsterdam, North Holland, Netherlands; Erasmus School of Health Policy & Management, Erasmus University Rotterdam, Rotterdam, Zuid-Holland, Netherlands; Internal Medicine, Section Geriatric Medicine, Amsterdam UMC Locatie Academic Medical Center, Amsterdam, Netherlands; Department of Medicine for Older People, Amsterdam UMC Location VUmc, Amsterdam, Noord-Holland, Netherlands; Department of Public Health Sciences, Penn State College of Medicine, Hershey, PA, USA

**Keywords:** long-term care reforms, interrupted time series analysis, nursing home admissions, survival, ageing-in-place, older people

## Abstract

**Background:**

The Netherlands introduced abrupt, large-scale, long-term care (LTC) reforms in 2015 that promoted ageing-in-place. However, there has been no comprehensive population-level study evaluating how these reforms have impacted nursing home (NH) utilisation. This study examines the association between the 2015 reforms with national monthly rates of NH admissions and survival time amongst newly admitted older adults.

**Methods:**

We analysed population data from Statistics Netherlands (2011–2019), conducting an interrupted time-series analysis to compare monthly NH admission rates before and after the 2015 reforms amongst adults aged 65 and older (N = 402 350). A Cox proportional hazards model was used to assess the reform’s impact on mortality risk amongst newly admitted residents.

**Results:**

The adjusted NH admission rate before the reform was 88.80 per 100 000 older adults (95% CI (confidence interval): 82.36–95.83), compared to 69.82 per 100 000 after the reform (95% CI: 65.91–73.78), indicating a significant reduction (incident rate ratio: 0.80, 95% CI: 0.74–0.86). Over a 3-year follow-up, the average survival time for those admitted after the reform was 608 days (95% CI: 608.72–610.74), compared to 622.52 days (95% CI: 620.59–624.45) for those admitted before the reform. The reform was associated with a slightly increased mortality risk (hazard ratio: 1.05, 95% CI: 1.02–1.07).

**Conclusions:**

The 2015 Dutch LTC reform is associated with a reduction in national NH admissions and a decrease in average survival time of 2 weeks.

## Graphical Abstract

**Graphical Abstract:**
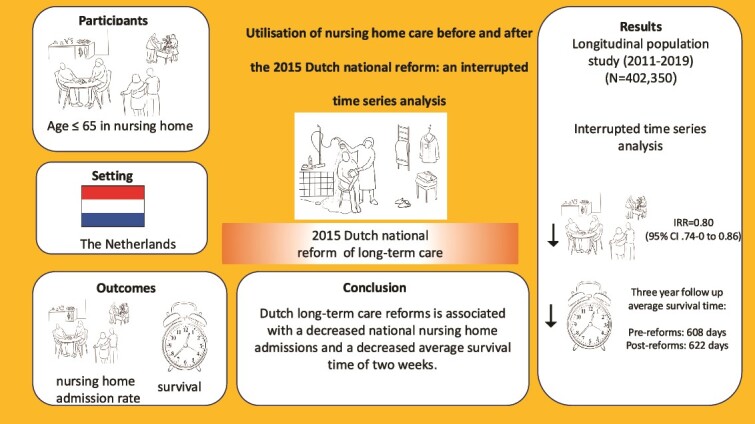

## Introduction

The Netherlands spends more on publicly financed long-term care (LTC) than any other country (3.1% of gross domestic product) [1]. Although these cross-country comparisons might be conflated in terms of definitions and scopes of LTC systems, the Netherlands has one of the most extensive LTC systems in the world, with a substantial amount allocated to older adults living in nursing homes (NH) [2]. Restricting access to institutional care to decrease costs began in 2013 with elimination of residential care for people with low care needs; however the Dutch government introduced a larger-scale reform on January 2015 that profoundly changed how social support and long-term care were organised [3]. The 2015 reform restricted NH care to only people that needed full-time, continuous care supervision. Both the 2013 and 2015 reforms promoted ageing-in-place [4], something many other countries have implemented in various forms [5, 6]. The Exceptional Medical Expenses Act of 2013 (Awbz) that covered long-term care at home plus NH care, was replaced on 1 January 2015 with multiple acts. The long-term care reforms of 2015 impacted services funded by the Social Support Act 2015 (Wmo 2015), community nursing under the Health Care Insurance Act (Zvw) and long-term care through the Long-term Care Act (Wlz) [3]. In addition to time spent in NH, other services affected included daycare, household assistance and home-based nursing and care. Short-term residential care, acute geriatric hospitals and waiting lists were engaged to further enable ageing-in-place. The 2015 Dutch LTC reform included the following key features: decreasing NH care; transfer of several social care services to family members and local community networks; decentralisation of social assistance; a transfer in funding of care at home from national LTC insurance to municipalities and health insurers; and spending cuts [4, 7, 8].

Although parts of the reform aimed at further reducing already declining NH admissions [9–12], the shift in financial responsibility for homecare and social assistance from the national level to municipalities and health insurers created a financial incentive for continued referral of individuals to NH care [8], which remained nationally financed. While cross-sectional evidence found decreased national NH spending in the reform year [13], other research reported increased NH admission amongst those municipalities at high risk of insolvency [11]. A population-level, longitudinal evaluation is needed to rigorously evaluate this reform’s association with subsequent NH utilisation because the reform impacts a large proportion of the population [3].

The aim of this study is to evaluate population-level, longitudinal data to compare the monthly rates of national NH admission and individual survival times of newly admitted residents before and after the Dutch 2015 reform. Survival time is a proxy for decreased length of stay as decreased survival suggests increased ageing-in-place. We hypothesised that in the post-reform period monthly rates of national NH admission and survival times would decrease.

## Methods

### Study design

We used an interrupted time series analysis (ITS) to evaluate the association of the Dutch LTC reform with the monthly rate of NH admission and survival time [14] from 2 January 2011 through 30 December 2019. ITS involves examining trends in the outcome of interest and approximating the change in trend after an intervention compared to the counterfactual [14]. The counterfactual scenario is the expected trend if the intervention had never occurred modelled from the pre-reform data [14]. This evaluation followed the RECORD statement [15].

### Study population and data

Using 9 years of population data from Statistics Netherland, we identified adults 65 years and older who were newly admitted for permanent NH. An independent assessment organisation (Centrum indicatiestelling Zorg) determined eligibility for permanent NH admission based on the interviewee’s current living condition, health, functional ability and well-being. This information is based on desk research, and is sometimes supplemented with a home visit and consultation that may include a doctor or other health care provider that knows the interviewee [16]. NH admission requires a care package indication between five to eight [16]. Care package five designates a person requiring intensive dementia care. Level six is for intensive personal care and nursing care [16]. Level seven is highly intensive care with a focus on supervision due to behavioural challenges and level eight is highly intensive personal care and nursing care targeted on activities of daily living and cognitive problems [16] (see Appendix 1 in the Supplementary data section for further details).

Data on NH admissions and discharges were extracted and linked with municipal records. There were some individuals with an unlikely admission date of January 1st (n = 10 488). To obtain a more accurate record of their admission dates, these individuals were linked to additional data reflecting their eligibility for NH admission. We aggregated the data to generate national estimates of monthly admissions and retained individual survival times for all residents.

### Primary outcomes of nursing home use

The two primary outcomes were the monthly rate of national NH admission and time from admission to death of each individual newly admitted. Because the goals and implementation of the 2015 Dutch Reform were focused on stricter entrance requirements for permanent NH stay, we restricted our focus to new admissions. A new admission required a minimal length of stay of 30 days between discharge and admission. We measured monthly admission rates for 4 years before (48 data points) and the 5 years after (60 data points) the 2015 Dutch LTC reform. This created a longitudinal record of each new admission to a NH facility. The monthly rate of national NH admission was determined by dividing the total number of new monthly NH admissions by the total monthly population of community dwelling adults 65 years and older. The second primary outcome was the days from admission to death for each new NH resident, assessing whether NH stay shortened post-reform period.

### Statistical analysis

Descriptive statistics summarised resident characteristics pre- and post-reform. Unadjusted yearly NH admission rates and survival times are in the appendix.

For the monthly rate of national NH admission, we assessed the incident rate ratio (IRR) for the 2015 Dutch LTC reform with an ITS analysis based on negative binomial (NB) regression. The NB distribution was chosen for its minimisation of the Bayesian information criterion (BIC) over other count distributions and offset was the log of the monthly population eligible for NH admission and all coefficients were estimated using robust error techniques. The multivariable model included: a binary indicator of reform, time in months, a measure of within year seasonality [17], and an interaction term between the reform and time variables (see Appendix 2 in the Supplementary Data section for further model details). We approximated the immediate step change in the monthly rate of admission by dividing the predicted rate for January 2015 over that from December 2014. The post-reform trend was calculated as the linear combination of the estimated pre-reform trend (time) and the change in trend (interaction term). Recommendations by Turner *et al.* [18] were used for graphing the IRRs, counterfactuals and seasonality.

Estimated survival medians, means and extended means in days were calculated for both study periods using individual level data. Kaplan Meier curves were also generated and Cox regression was used to model time from admission to death using Efron’s method for ties based on robust error estimates. The proportional hazards assumption was checked with the link test [19], by testing Schoenfeld residuals and by plotting the proportional hazards curves. The model included a binary indicator of reform, age at admission, sex, care package level at admission and year of admission. Due to covariate-related issues of convergence, restricted mean survival time over a period of 3 years was calculated without adjustment [20]. All analyses were conducted in Stata 16.0 with statistical significance defined as a two-tailed *P* < 0.05.

### Sensitivity analyses

We conducted a number of sensitivity analyses to test the robustness of our findings. The first of these tested whether there were meaningful breaks in the time series other than our focus of interest, i.e. the reform initiated in 2015. These included checking whether the elimination of care for persons with low care needs that started in January of 2013 registered as a notable break using a Chow test that performs linear regression of time on the observed rates. We also checked how exclusion of specific intervals of data that may have been temporally confounded with other changes affected our outcomes. These tests included re-fitting our final model to data that excluded all data prior to 2013 which had included some unexplained missingness and separately re-fitting the model to data that excluded only the year the reform was implemented. The latter case tested how the development of new services in the year of implementation affected our results. To further test how the elimination of low-level care that commenced in January of 2013 affected our results, we re-fit our model after re-labelling the intervention as starting in January 2013 rather than in January of 2015. Finally, in order to assure ourselves that the robust standard errors in our negative binomial regression of the rates sufficiently addressed the serial correlation of the sequential monthly rates, we added an autoregressive explanatory term consisting of the preceding months rate to our model. For the survival analysis, the BIC was used to test whether addition of the following interactions improved model fit: time to event by sex, time to event by age and sex by age.

## Results

### Population characteristics

Table 1 presents population charachteristics. Unadjusted national monthly NH admission rates were significantly lower post-reform than pre-reform (Table 1). The unadjusted yearly rates of national NH admission are presented in the Appendix.

**Figure 1:**
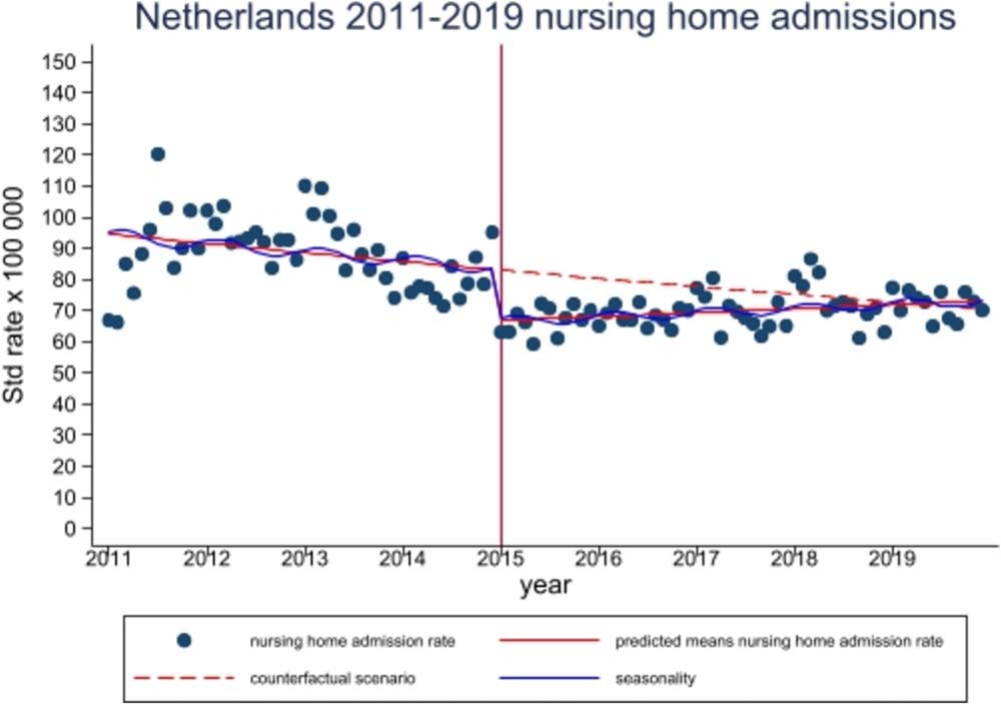
Monthly rate of nursing home admission per 100 000 Dutch adults ages 65 years and older, January 2009–December 2019 (N = 402 350). The dots show the observed rate of monthly nursing home admissions per 100 000 older adults. The solid line shows the rate of NH admissions estimated by the adjusted reform model (negative binomial regression modelling). For example, in June 2011 the adjusted reform model estimated that there were 92.91 NH admissions per 100 000 older adults. The dashed line shows the counterfactual that estimated the rate of NH admissions if no reform occurred (negative binomial regression modelling). E.g. for Feb 2019 the counterfactual estimated that there would be 72.60 NH admissions per 100 000 older adults. In contrast, the adjusted reform model estimated that in Feb 2019 there would be 73.48 admissions per 100 000 older adults (solid line). This finding suggests that the rate of monthly NH admissions estimated by the adjusted reform model surpasses that estimated by the counterfactual by early 2019.

**Table 1:** Population cohort characteristics, 2011–19.

	Pre-reform (N = 194 559)	Post-reform (N = 207 791)	*P*-value
Patient level characteristics			
Age, years	87 [82–91]	86 [81–91]	<0.0001^*^
Sex			
Female	130 363 (67.00%)	133 577 (64.28%)	<0.0001^†^
Male	64 196 (33.00%)	74 214 (35.72%)	
Care dependency level at admission^a^			<0.0001^†^
5	100 148 (51.47%)	132 137 (63.59%)	
6	77 332 (39.75%)	69 617 (33.50%)	
7	12 124 (6.23)	1973 (1.96%)	
8	4955 (2.55)	1973 (0.95%)	
Nursing home level characteristics			
Monthly number of nursing home admission Mean (SD)	4053 (509)	3463 (315)	<0.0001^*^
Monthly nursing home admission rate^‡^ Mean (SD)	88.77 (11.61)	69.84 (5.66)	<0.0001^*^

Data are median [IQR], n (%) or mean (SD). ^*^Independent t-test, ^†^Chi-square. ^‡^Monthly nursing home admission rate = (monthly nursing home admissions/monthly standardised total population 65 years and older) × 100 000.

^a^Level five is specific to persons with dementia that require intensive dementia care [24]. Level six is for intensive personal care and nursing care [24]. Level seven is care with highly intensive care with a focus on supervision due to behavioural challenges and level eight is highly intensive care focused on personal care and nursing care that targets activities of daily living and cognitive problems [24].

In the pre-reform period, the monthly rate of national NH admission did not significantly change over time [IRR 0.997 (95%CI 0.994–1.001, *P* = 0.130); Figure 1; Table 2]. The adjusted pre-reform rate per 100 000 older adults was 88.80 (95%CI 82.36–95.83) whereas the adjusted post-reform rate was 69.82 (95 CI% 65.91–73.78), reflecting a decreasing effect of the reform [IRR 0.80 (0.74–0.86, *P* < 0.0001)]. The step change in the monthly admission rate immediately following implementation, from December 2014 to January 2015, was a decrease of 19.5%. This was followed by a positive trend change with an IRR of 1.004 (95%CI 1.001–1.008, *P* = 0.017) that resulted in an increasing trend over the post-reform period: IRR of 1.002 (95%CI 1.001–1.003, *P* < 0.001). This trend was equivalent to an increase of 0.2% in national NH admission per month. Because of the increasing trend in the post-reform period, the monthly NH admission rate predicted by the multivariable model was higher than that predicted by the counterfactual model by February 2019 (see Figure 1).

**Table 2:** Incident rate ratio of monthly rate of national nursing home admission from interrupted time series analysis (N = 402 350) adjusted for seasonality.

Model Variable	Nursing home admission rate IRR (95% Confidence interval); *P*-value
Average reform effect (${\beta}_1$Reform)	0.795 (0.735–0.859), *P* < 0.0001
Pre-reform trend (${\beta}_2$Time)	0.997 (0.994–1.001); *P* = 0.130
Trend change (Interaction between Reform and pre-reform trend (${\beta}_3$)	1.004 (1.001–1.008), *P* < 0.017
Post intervention trend (linear combination of pre-reform trend and trend change)	1.002 (1.001–1.003); *P* < 0.001

IRR = incident rate ratio.

### Changes in length of stay

**Figure 2:**
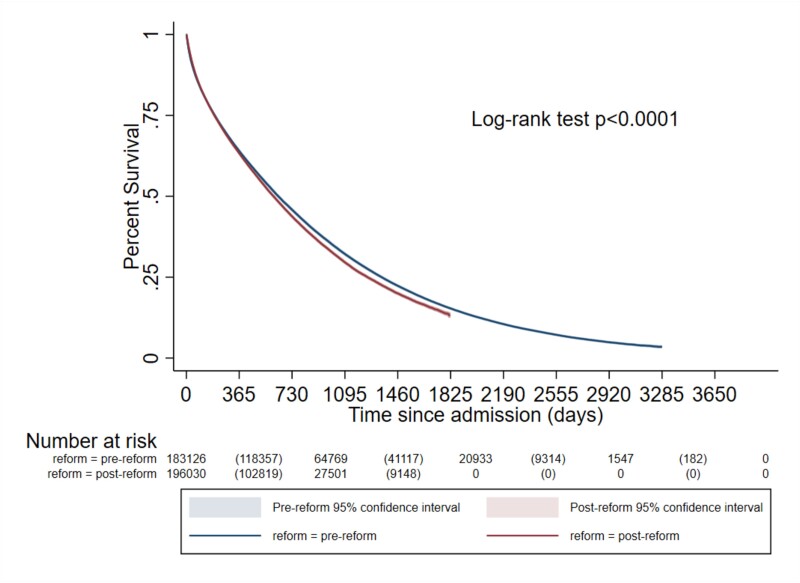
Kaplan Meier survival estimates in relation to time since admission for older adults 65 years and older newly admitted to nursing homes before the 2015 Dutch Long-term care reform and after the reform (Log-rank test *P* < 0.0001).

The Kaplan–Meier (Figure 2) curves illustrated lower survival probability in the post-reform period (log-rank test for equality of survivor function *P* < 0.0001). The multivariable Cox model of time to death demonstrated that residents admitted to NHs in the post-reform period had higher hazards of death than those admitted in the pre-reform period [hazard ratio 1.05 (1.02–1.07)]. Over 3 years of follow-up, the post-reform restricted mean survival time was 608 days [608.72–610.74] versus 622.52 days [620.59–624.45] in the pre-reform period (Table 3).

**Table 3:** Estimated nursing home length of stay in days using the median, mean, extended mean and restricted mean at 3 years from admission to death of each individual newly admitted older adults 65 years and older from statistical models.

	Pre-reform	Post-reform	Total
	183 126	196 030	379 156
Median	635 (630–640)	601 (597–607)	619 (616–623)
Mean	904.28 (900.22–908.33)	758.08 (754.75–761.42)	888.15 (885.00–891.31)
Extended mean	938.30	877.73	
Restricted mean at 3 years	622.52 (620.60–624.45)	608.74 (606.74610.74)	

### Sensitivity analysis

We performed sensitivity analyses, which confirmed the robustness of our results; details are in the appendix.

## Discussion

This is the first study to longitudinally evaluate the effect of the Dutch 2015 LTC reform on NH utilisation using population-level data. NH utilisation, as measured by monthly national admission rates and individual survival time, was significantly lower in the post-reform period than the pre-reform period. The results suggest that the monthly rate of national NH admission decreased dramatically right after the 2015 Dutch LTC reform but the rates started to increase over time. The reform’s effect on admission rates appears temporary; by the end of our observation period, the admission rate was similar to the counterfactual rate predicted based on pre-reform admission trends. Our results were robust in testing for sensitivity to the smaller 2013 reform. In an adjusted model, residents admitted in the post-reform period had five percent higher hazard of death than those admitted in the pre-reform period and, on average, stayed two weeks less in NH facilities (609 days in the post-reform versus 623 in the pre-reform period). Because the reform was intended to restrict access to the most severe cases, we hypothesised decreases in the monthly rate of national NH admission and in time to death during the post-reform period. Contradicting the aims of the Dutch reform, in the post-reform period we observed an increased proportion of older adults admitted to NH who were younger (65 ≤ 80) and a higher proportion of patients admitted with dementia in the lowest care level (care package five).

MacNeil Vroomen *et al.* [21] used ITS to evaluate changes in location of death of older adults after the implementation of the Dutch 2015 LTC reform and found that proportions of death at home and hospital increased over death in NH. This is consistent with our findings of decreased NH admissions after the reforms. After the 2015 Dutch reform, home became the primary location of death for the Dutch older adult population [21]. MacNeil Vroomen *et al.* [21] also found that NH were the primary location of death amongst persons with dementia and that their proportion of death at home did not change, even as overall risk of hospital death increased. Our current results extend this population-level work by examining LTC utilisation.

Our results are consistent with the findings of Krabbe-Alkemade *et al.* [13] who found that in the year after the 2015 Dutch LTC reform was implemented, NH utilisation decreased from the preceding year. Our study adds to the longitudinal evidence by demonstrating there was a dramatic decrease in the monthly rate of admission associated with the 2015 reform and that over the first 4 years of the post-reform period, this rate increased until exceeding that predicted by the slope of the pre-reform period.

Our study has several notable limitations. We excluded residential care from our analysis because pre- 2015 data did not distinguish between older adults in residential care and those receiving homecare. Additionally, pre-reform NH admission rates showed variability, which we could not explain. We tried to correct this by including quadratic and cubic terms for time, however the BIC showed this did not help our model. Another limitation is the strong stationarity assumptions in sensitive tests. ITS assumes that trends can be completely factored out and that means and variances are stable over time. For example, the unknown structural break test found November 2013 to be a structural break in our time series data; however, given that no major reforms occurred during that period, this was puzzling. Simple linear tests such as the Chow test have strong stationarity assumptions that are not met by our data and that may result in the detection of spurious associations. We also cannot explain why younger men are more likely to be admitted to NHs in the post-reform period. Finally, our study does not consider any reform-related effects on the well-being of informal caregivers.

There is growing evidence that older adults were impacted after the 2015 Dutch LTC reform. Our results showed NH admission priority to adults with dementia with the lowest care package compared to the rest of the older adult population. This might have limited options for older adults who may have wanted to be institutionalised. Evidence of the change in care includes one study that found an increase in hospital utilisation the year of the reform [22]. Other researchers found that as part of the reforms most patients were admitted into short-term residential care on a transitional basis until discharge home, death, or transfer to NH facilities [23]. Other research found an increase in hospital deaths for persons with and without dementia [21]. Most profoundly home was identified as the primary location of death amongst older adults [21], which may indicate informal care utilisation, homecare or primary care. A Cochrane review found insufficient evidence for supporting home-based alternatives to institutional care for older adults [24]. From a patient perspective, ageing-in-place policies can also leave this patient population vulnerable to fragmented care [25], inconsistent informal care [26], and social inequity to accessing services [27].

Fewer older adults in nursing homes leave general practitioners managing more complex patients, often with elderly care physicians and fragmented care coordination [3, 28]. Since the 2015 reform, health care professionals may need to rely more on informal caregiving, intermediate care, homecare and acute beds which some general practitioners have found to be more costly [13, 23, 29].

From a payer’s perspective, we found that older adults admitted during the post-reform period stayed 14 fewer days in NH compared to the pre-reform period. Using the 2022 standardised average daily LTC cost of 290 euros per resident, the savings is roughly 4205 euros per resident [30]. If we assume that the reduction in admissions was temporary but the reduction in length of stay permanent, we can multiply this number by the 151,770 residents in NH to calculate an estimated annual savings of 638 million euros [31]. Nevertheless, part of these savings will be discounted by additional care and social assistance provided at home [32]. Policy studies of similar changes in NH stays suggest that between 50 and 60% of costs savings will be offset by increased costs of care at home [32, 33]. A previous study [34] in the Netherlands on the postponing of NH admission for low care intensity packages found that increases in costs at home resulted in little overall savings.

Previous authors [4] reported on the confusion and upheaval based on the first year of the reform across homecare, hospitals, intermediate care, retirement homes that municipal and regional governments, older adults, informal caregivers and health care professionals all had to contend with. The reform was ambitious in both size and scope. It included a drastic change in the organisation of long-term care, dividing the responsibilities that fell under a single social insurance over three different entities. Especially for the over 300 Dutch municipalities, this entailed fulfilling a new role with which they had little experience. At the same time, budget cuts were implemented [4] and municipalities had to contend with a frailer population as result of the restricted access to institutional care prior and ruing the reform. Several papers [11, 35] showed municipalities that were at high risk for insolvency or otherwise restricted in their ability to finance the homecare of residents experienced increased NH admission in the post-reform period. An independent evaluation by the Netherlands Institute for Social Research found that the reforms only were partly successful [12]. It also seems that many of the original budget cuts were reversed in later years [36]. Together with our finding that admission rates in the longer run returned to the already decreasing trend prior to the reform, this suggests that a more staggered implementation of the reform might have been beneficial. It seems likely that similar long-run outcomes might have been achieved without the negative transitional costs of the abrupt implementation of the reform.

We conclude that the reform appears to have temporarily accelerated the ongoing decline in NH utilisation. For future reforms that promote ageing-in-place, a more gradual implementation, combined with continuous systematic assessment, is strongly recommended to reduce unnecessary transitional costs. As the Netherlands is the world’s largest spender on LTC care [1], and has implemented extensive systemic reform [4], it serves as a valuable case study for other countries. Future research should focus on developing comprehensive frameworks to evaluate the effectiveness of reform policies that promote ageing-in-place.
